# Disordered gambling in a longitudinal birth cohort: from childhood precursors to adult life outcomes

**DOI:** 10.1017/S0033291722003051

**Published:** 2023-09

**Authors:** Wendy S. Slutske, Leah S. Richmond-Rakerd, Thomas M. Piasecki, Sandhya Ramrakha, Richie Poulton, Terrie E. Moffitt, Avshalom Caspi

**Affiliations:** 1 Department of Family Medicine and Community Health, University of Wisconsin School of Medicine and Public Health, Madison, WI, USA; 2 Department of Psychology, University of Michigan, Ann Arbor, MI, USA; 3 Department of Medicine, University of Wisconsin School of Medicine and Public Health, Madison, WI, USA; 4 Department of Psychology, University of Otago, Dunedin, New Zealand; 5 Department of Psychology and Neuroscience, Duke University, Durham, North Carolina, USA

**Keywords:** course, crime, disordered gambling, Dunedin longitudinal birth cohort, incidence, recurrence, self-control, stability

## Abstract

**Background:**

Despite its introduction into the diagnostic nomenclature over four decades ago, there remain large knowledge gaps about disordered gambling. The primary aims of the present study were to document the long-term course, childhood precursors, and adult life outcomes associated with disordered gambling.

**Methods:**

Participants enrolled in the population-representative Dunedin Study were prospectively followed from birth through age 45. Disordered gambling was assessed six times from age 18; composite measures of childhood social class, general intelligence, and low self-control were based on assessments obtained from birth through age 15; adult socioeconomic, financial, and legal outcomes were obtained through age 45. Lifetime disordered gambling was predicted from the three childhood precursors and the adult outcomes were predicted from lifetime disordered gambling.

**Results:**

Past-year disordered gambling usually occurred at only a single time point and recurrence was relatively uncommon. Lower childhood social class, general intelligence, and self-control significantly predicted lifetime disordered gambling in adulthood. In turn, lifetime disordered gambling in adulthood significantly predicted occupational, educational, and financial problems in adulthood (*ds* = 0.23–0.41). These associations were markedly reduced and sometimes rendered nonsignificant after adjusting for childhood precursors (*ds* = 0.04–0.32).

**Conclusions:**

Socioeconomic, financial, and legal outcomes in adulthood are not merely consequences of disordered gambling, but also are predicted from childhood precursors. Deflecting the trajectories of young people at risk for developing disordered gambling may help to ameliorate not just the development of later disordered gambling, but also other associated adverse outcomes.

## Introduction

Despite its introduction into the diagnostic nomenclature over four decades ago (APA, 1980; WHO, 1977), there remain large knowledge gaps about the long-term course and outcomes associated with gambling disorder. Studies of individuals who have sought treatment have demonstrated that gambling disorder is associated with adverse life outcomes, particularly financial and legal problems (Lesieur, 1998; Potenza et al., 2019), but because very few individuals with a gambling disorder seek treatment (Slutske, 2006), the results of studies based on this minority may not generalize to the larger population of those with gambling disorder in the community.

Evidence from cross-sectional research suggests that the most common course of gambling disorder is a single episode lasting one year or less (Slutske, 2006), and a handful of longitudinal studies have yielded findings that are consistent with this (LaPlante, Nelson, LaBrie, & Shaffer, 2008; Scherrer et al., 2007; Slutske, Jackson, & Sher, 2003). However, there is no longitudinal research that has documented the longer-term course of disordered gambling*^1^ and the extent to which new onsets are observed beyond the fourth decade of life. In addition, the distinction between episodic and persistent gambling disorder was introduced into the most recent revision of the DSM; but to our knowledge, there have been no studies comparing individuals with an episodic or persistent course of disorder.

Nearly all previous longitudinal studies have included aspects of low self-control as risk factors for disordered gambling (Dowling et al., 2017), but few prospective studies have focused on the relation between disordered gambling and other established risk factors for psychiatric disorders, such as general intelligence or family social class. For example, socioeconomic disadvantage has been considered a consequence of gambling disorder, given the devastating losses of money, time, and opportunities that can occur (Fong, 2005). Indeed, gambling-related financial losses are pathognomonic and are a diagnostic criterion for DSM-5 gambling disorder. However, socioeconomic disadvantage may also be a risk factor for gambling disorder. Socioeconomically disadvantaged individuals may see gambling as a form of investment and a possible escape from poverty (Welte, Barnes, Wieczorek, Tidwell, & Parker, 2004), those from the higher social classes may be able to gamble the same amount without suffering the same consequences as their less fortunate counterparts (Welte et al., 2004), and poverty may impair psychological processes such as cognition and decision making (Mani, Mullainathan, Shafir, & Zhao, 2013). The extant literature based on cross-sectional studies cannot adjudicate between these two alternate interpretations of the association between socioeconomic status and gambling disorder. Prospective longitudinal studies that are initiated prior to the onset of the gambling career are needed to differentiate the potential consequences from the potential causes of disordered gambling.

The aims of the present study were to (1) document the long-term course of disordered gambling through age 45 in a population-representative longitudinal birth cohort, (2) examine childhood precursors and important life outcomes in adulthood associated with disordered gambling, and (3) compare the childhood precursors and adult outcomes of cohort members with episodic and persistent disordered gambling. The extremely low rates of attrition in the Dunedin Study (the present study sample) provided a rare unimpeded view of the course and consequences of disordered gambling through age 45 in a complete birth cohort. Importantly, because cohort members were prospectively followed since birth, it was possible to determine whether any associations observed between disordered gambling and important life outcomes in adulthood represented potential consequences of disordered gambling or were explained by pre-existing differences in childhood.

## Methods

### Participants

Participants are members of the Dunedin Multidisciplinary Health and Development Study, a longitudinal investigation of health and behavior in a complete birth cohort. The cohort of 1037 children (52% male) was constituted at age 3 when the investigators enrolled 91% of consecutive births between 1 April 1972 and 31 March 1973 in Dunedin, New Zealand. The cohort represented the full range of socioeconomic status in the general population of New Zealand's South Island. The cohort is primarily white (~93%), matching South Island demographics. Assessments were held at birth and at ages 3, 5, 7, 9, 11, 13, 15, 18, 21, 26, 32, 38, and, most recently, 45 years, when 938 of the 997 living cohort members (94%) took part. [For more information about the Dunedin cohort see online Supplemental Materials and (Poulton, Moffitt, & Silva, 2015)].

### Measures

#### Disordered gambling

Diagnostic assessments of past-year disordered gambling were administered via structured face-to-face interviews conducted at ages 21, 32, and 45 that differed at the three ages. At age 21, the disordered gambling assessment comprised eight items from the South Oaks Gambling Screen (SOGS; Lesieur and Blume, 1987; see Slutske et al., 2005, for more details). At age 32, it was based on two different gambling assessments: the Sydney Laval Universities Gambling Screen (SLUGS; Blaszczynski, Ladouceur, and Moodie, 2008) and the National Opinion Research Center DSM-IV Screen for Gambling Problems (NODS; Gerstein et al., 1999; seeSlutske et al., 2012, for more details). At age 45, it was based on two different gambling assessments: Problem Gambling Severity Index (PGSI; Ferris and Wynne, 2001) and the NODS (Gerstein et al., 1999). Those who reported that they had spent $100 or more on gambling in the past year were administered the PGSI (40.2% of the sample), and among those who were administered the PGSI, those who endorsed at least one symptom were administered the NODS (2.0% of the sample).

The past-year prevalences of DSM-5 gambling disorder were low [2.09% and 0.66% at ages 32 and 45, respectively (DSM-5 diagnostic criteria were not assessed at age 21)]; therefore, we focused on a broad definition of disordered gambling.

Because the diagnostic assessments varied at ages 21, 32, and 45, we derived a 5-item harmonized measure of ***past-year disordered gambling*** to maximize cross-wave comparability. This included the symptoms of financial problems, chasing losses, spending more time or money than intended, borrowing money, and hiding evidence or lying about gambling (see online Table S1 in Supplemental Materials). The internal consistency reliabilities of the 5-item harmonized disordered gambling measures were generally acceptable (age 21: *α* = 0.58; age 32: *α* = 0.87; age 45: *α* = 0.79), and their strong cross-wave associations suggested that they were measuring the same construct (gamma _21/32_ = 0.66; gamma _21/45_ = 0.70; gamma _32/45_ = 0.91, all *p*s < 0.0001; Goodman and Kruskal, 1954).

There was also a single item about past-year disordered gambling included in a delinquency assessment that was administered at ages 18, 26, 32, 38, and 45, with identical items used for all ages but 18 (‘In the past year, has gambling or betting ever been a problem for you? For example, have you bet or gambled money your family needed? Borrowed money, sold property or gone into overdraft to pay for gambling?’).

A combined diagnosis of ***lifetime disordered gambling*** was based on a diagnosis of past-year disordered gambling at any of the eight assessments obtained at six ages from 18 to 45, that is, the harmonized measure available at three Phases (21, 32, 45), and the single-item measure from the delinquency assessment available at five Phases (18, 21, 26, 32, 38, and 45). Given the low prevalence of past-year disordered gambling, this combined diagnosis was used in the regression analyses focused on precursors and outcomes associated with lifetime disordered gambling. A lifetime disordered gambling diagnosis was made if there were data available from at least three Phases (*N* = 975).

The different self-report measures of disordered gambling, that is, the harmonized measure of past-year disordered gambling, the single item from the delinquency assessment (both at age 45), and the lifetime disordered gambling measure were validated by examining their associations with a measure of gambling involvement and an alternate measure of problems obtained at age 45.

The measure of gambling involvement was a count of the number of 11 different gambling activities in the past year, referred to as ‘gambling versatility.’ Past-year harmonized disordered gambling (*d* = 1.53, *p* < 0.0001) and the single-item assessment at age 45 (*d* = 1.06, *p* < 0.0005) and lifetime disordered gambling (*d* = 0.87, *p* < 0.001) were all significantly associated with past-year gambling versatility.

The alternate measure of gambling problems was based on reports from three informants (e.g. best friend, partner, or other family member) nominated by each study member. Informants rated the study member on the item ‘has problems with gambling’ using a three-point scale, and responses were averaged across informants. Past-year harmonized disordered gambling (*d* = 2.37, *p* < 0.0001), the single-item assessment (*d* = 4.13, *p* < 0.0001) at age 45 and lifetime disordered gambling (*d* = 0.70, *p* < 0.001) were all significantly associated with informant reports of gambling problems.

#### Childhood background factors

The composite measures of childhood social class, general intelligence, and self-control included in this study (Moffitt et al., 2011; Richmond-Rakerd et al., 2021) are described in the Supplemental Materials [text and online Supplementary Table S2].

#### Life outcomes

We analyzed potential outcomes associated with disordered gambling in three domains: socioeconomic (occupational attainment, educational attainment), financial (credit scores, unemployment, social welfare benefits, practical financial knowledge, informant-reported financial problems) and legal (adult criminal convictions). Financial loss is considered by some to be the fundamental consequence of gambling problems from which other consequences, such as engaging in criminal activities, follow (Adolphe, Khatib, van Golde, Gainsbury, & Blaszczynski, 2019; Ladouceur, 2004). In addition to self-report, many of the outcomes were assessed using administrative records and informant reports. This may be especially important for a study of disordered gambling because there can be concerns about the veracity of self-reports; for example, a DSM-5 symptom of gambling disorder is ‘lies to conceal the extent of involvement with gambling.’ Descriptions of the life outcome assessments are presented in online Table S3 in the Supplemental Materials.

### Data analysis

#### Stability and course of past-year disordered gambling

Mean-level stability was examined by comparing the prevalences of harmonized and single-item past-year disordered gambling across six waves. The single-item measure was identical at ages 26, 32, 38, and 45, so was the best indicator with which to make a cross-wave comparison. In addition to estimating the prevalence of disordered gambling, we also estimated the incidence, or the onset of new cases of disordered gambling, at ages 21, 26, 32, 38, and 45. Intra-individual stability was examined by classifying each participant into discrete groups based on their pattern of harmonized disordered gambling diagnoses at ages 21, 32, and 45. Given the small sample sizes and corresponding lack of statistical power, these analyses were primarily descriptive.

#### Childhood precursors associated with disordered gambling

These associations were examined using logistic regression. Potential sex differences were evaluated by incorporating a childhood precursor-by-sex interaction in the model; none of these interactions were statistically significant (all *ps* > 0.15) and therefore we combined men and women in all analyses (but controlled for sex).

#### Life outcomes associated with lifetime disordered gambling

These associations were examined using linear or logistic regression. One set of analyses include only sex as a covariate and a second set included sex and the three childhood background factors as covariates. Potential sex differences were evaluated by incorporating a disordered gambling-by-sex interaction in the model; none of these interactions were statistically significant (all *ps* > 0.25) and therefore we combined men and women in all analyses (but controlled for sex).

#### Comparisons of single episode and recurrent disordered gambling

Study members who were diagnosed with disordered gambling at only one of the six study waves^2^ or at more than one study wave were considered to have ‘single episode’ or ‘recurrent’ disordered gambling, respectively. These terms better suited the patterns of diagnoses in these data than did the specifiers of ‘episodic’ and ‘persistent.’ Because the past-year assessments were conducted 3–5 years apart, it was not possible to characterize study members as having a persistent course and all participants would have been characterized as having an episodic course. The linear and logistic regressions were repeated using dummy codes to specify whether study members had experienced zero, one, or more than one episode of disordered gambling across six waves of the study, with the critical test of interest being the contrast between more than one and one episodes.

## Results

### Stability and course of disordered gambling

There was a general trend for the prevalence of disordered gambling to decrease from age 21 to age 45 (Table 1). This was apparent for harmonized disordered gambling (Table 1, row 3), and the single-item disordered gambling assessment (Table 1, row 2). Gambling involvement also decreased from ages 21 to 32 to 45 (Table 1, row 1). The incidence of disordered gambling decreased from age 21 to 45; nonetheless, nearly a third of the cases of disordered gambling at age 45 (29%) were incident cases (Table 1, row 4).

**Table 1.** Prevalences of any gambling, disordered gambling, and disordered gambling incidence at six study phases

		Study phase (age)											
row	Gambling outcome	18		21		26		32		38		45	
		*n* = 836		*n* = 939		*n* = 977		*n* = 961		*n* = 960		*n* = 908	
		%	*n*	%	*n*	%	*n*	%	*n*	%	*n*	%	*n*
1	Any gambling	–	–	86.47	812	–	–	79.08	760	–	–	77.20	701
2	Single item disordered gambling^a^	1.32	11	–	–	1.94	19	1.98	19	1.48	14	1.20	11
3	Harmonized disordered gambling^b^	–	–	13.31	125	–	–	5.85	56	–	–	3.19	29
4	Disordered gambling incidence^c^	–	–	95	119	53	10	52	29	21	3	29	8

a Item was identical at all phases but 18.

b Harmonized assessment included the following five symptoms: financial problems, chasing losses, spending more time or money than intended, borrowing money, hiding evidence or lying about gambling.

c Percentage of cases in rows 2 (Phases 18, 26, and 38), 3 (Phase 21) or either rows 2 or 3 (Phase 32 and 45) that were new cases (not diagnosed in any previous waves).

There was low intra-individual stability of disordered gambling. Of those who experienced at least one episode of harmonized disordered gambling, 78.4% experienced just a single episode (Table 2)^3^. Among those who experienced multiple episodes of harmonized disordered gambling, 84.4% experienced two episodes and 15.6% experienced disordered gambling at all three waves. A similar pattern was observed for the single-item disordered gambling assessment (online Supplementary Table S4). [Note that non-disordered gambling involvement evidenced much greater intra-individual stability (online Supplementary Table S5)].

**Table 2.** Course of harmonized disordered gambling across three study phases

	Full sample	1 or more episodes		Study phase (age)				
	%	%	*n*	21	32	45	%	*n*
No disorder	82.75	–	710	−	−	−	82.75	710
Single episode	13.52	78.38	116	+	−	−	10.14	87
				−	+	−	2.45	21
				−	−	+	0.93	8
Recurrent/persistent	3.73	20.95	32	+	+	−	1.40	12
				+	−	+	0.82	7
				−	+	+	0.93	8
				+	+	+	0.58	5

*Note*: Harmonized assessment included the following five symptoms: financial problems, chasing losses, spending more time or money than intended, borrowing money, hiding evidence or lying about gambling; minus = absence of disordered gambling, plus = presence of disordered gambling. For comparison, the course of any gambling involvement is presented in online Table S5 in the Supplemental Materials.

In contrast to the low *intra-individual* stability, the stability of individual differences, that is, *inter-individual* stability of disordered gambling (Slutske, 2007) was remarkably high. Gamma coefficients ranged from 0.60 to 0.97 (mean = 0.84), with an impressive inter-individual stability of 0.70 across a time span of 24 years (online Supplementary Table S6).

### Childhood precursors of disordered gambling

**Fig. 1.**
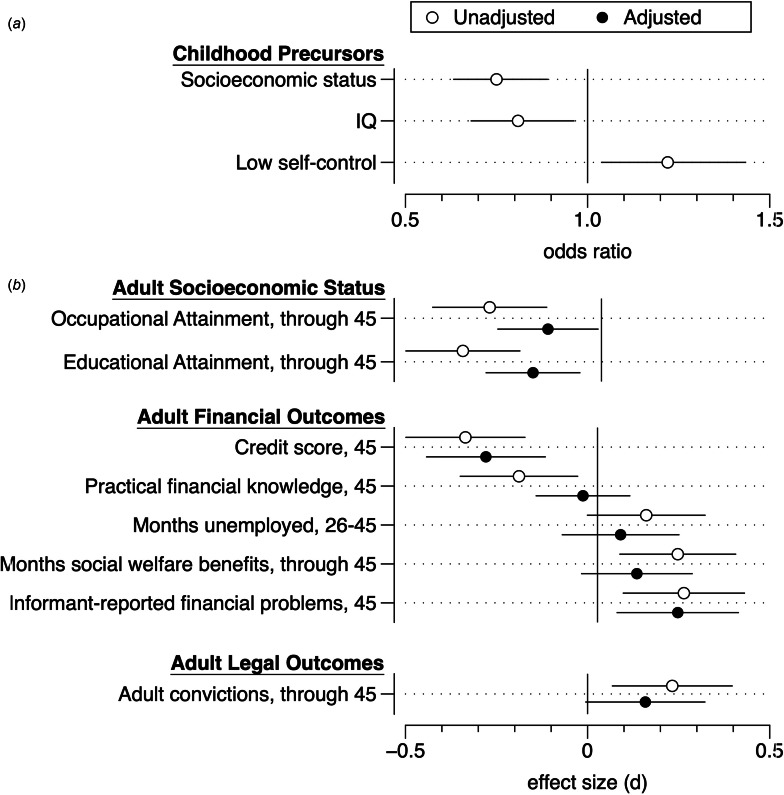
Associations of childhood precursors (panel *a*) and adult outcomes (panel *b*) with lifetime disordered gambling. Unadjusted associations control for sex, adjusted associations control for sex, childhood socioeconomic status, childhood IQ, and childhood low self-control. Horizontal bars are 95% confidence intervals.

Childhood socioeconomic status [odds ratio (OR) 0.75, 95% confidence interval (CI) 0.63–0.89, *p* = 0.001], general intelligence [OR 0.81 (CI 0.68–0.96), *p* = 0.018], and low self-control [OR 1.22 (CI 1.04–1.44), *p* = 0.017] all significantly predicted lifetime disordered gambling by age 45 (see Table 3 and Fig. 1). These childhood background factors also predicted the adult outcomes (see online Table S7 in Supplemental Materials).

**Table 3.** Life outcomes at age 45 associated with lifetime disordered gambling^a^

Variable and source^b^		Disordered gambling	No disordered gambling	Adjusted for sex		Adjusted for sex, childhood SES, IQ, and self-control^b^	
		*N* = 180	*N* = 795	Odds ratio	*p*	Odds ratio	*p*
Sex				2.92	<0.001	–	–
Male		129 (25.90%)	369 (74.10%)				
Female		51 (10.69%)	426 (89.31%)				
Childhood Background Factors							
Childhood socioeconomic status, 0–15	P	3.51 (1.02)	3.82 (1.15)	0.75	0.001	0.78	0.010
Childhood IQ, 7–11	L	98.71 (14.74)	101.04 (14.00)	0.81	0.018	0.95	0.652
Childhood low self-control, 3–11	M	0.24 (1.04)	−0.07 (0.93)	1.22	0.017	1.09	0.350
				*b*	*p*	*b*	*p*
Socioeconomic							
Occupational attainment, 45	S	3.29 (1.54)	3.83 (1.45)	−0.49	<0.001	−0.23	0.038
Educational attainment, 45	S	1.50 (1.06)	1.96 (0.98)	−0.41	<0.001	−0.20	0.005
Financial							
Credit score^c^, 45	R	701.18 (178.09)	762.74 (138.21)	−56.10	<0.001	−47.40	<0.001
Practical financial knowledge, 45	L	−0.11 (0.96)	0.02 (0.90)	−0.21	0.009	−0.04	0.547
Months unemployed, 26–45	S	4.39 (12.29)	2.89 (9.68)	1.44	0.107	0.68	0.441
Months social welfare benefits, through 45	R	38.00 (63.54)	26.86 (56.76)	13.53	0.007	6.63	0.167
Informant reported financial problems, 45	I	0.53 (0.72)	0.37 (0.58)	0.15	0.006	0.14	0.010
Legal							
Adult convictions, through 45	R	4.77 (12.13)	1.93 (8.75)	2.20	0.006	1.50	0.059

S, self-report; R, administrative record; P, parent report; I, informant report; L, laboratory; M, multi-modal.

a This included both the harmonized and single-item assessments of disordered gambling that were used separately in the stability analyses reported in Table 2 and online Supplementary Table S4.

b For the three childhood background factors, adjusted for the other two.

c Six study members were flagged by Equifax/Veda as insolvent at phase 45 and were assigned a score of 66 (one point less than the lowest score among study members with a credit score).

### Life outcomes associated with lifetime disordered gambling

Lifetime disordered gambling significantly predicted lower occupational [*d* = −0.33 (CI −0.50 to −0.16), *t* = −3.82, df = 1, *p* < 0.001] and educational attainment [*d* = −0.41 (CI −0.58 to −0.24), *t* = −4.73, df = 1, *p* < 0.001]. It was also associated with having a lower credit rating [*d* = −0.38 (CI −0.56 to −0.21)], spending more time on social welfare benefits [*d* = 0.23 (CI 0.06–0.40)], having less practical financial knowledge [*d* = −0.23 (CI −0.40 to −0.06)], and more informant-reported financial problems [*d* = 0.25 (CI 0.07–0.43)]. Lifetime disordered gambling also predicted more adult criminal convictions [*d* = 0.23 (CI 0.07–0.43)]. Four of these seven significant associations survived adjustment for childhood background factors (occupational and educational attainment, credit rating and informant-reported financial problems; see Table 3 and Fig. 1), and only two (educational attainment and credit rating) survived adjustment after imposing a Bonferroni correction for multiple testing (i.e. 0.05/8 = 0.006).

### Comparisons of single episode *v.* recurrent disordered gambling

Of the 180 study members with a diagnosis of lifetime disordered gambling, 72% (*n* = 129) experienced a single episode and 28% (*n* = 51) had experienced more than one; that is, they had recurrent disordered gambling. With respect to the three childhood precursors, those with lower childhood socioeconomic status were more likely to have recurrent than single-episode disordered gambling [OR 1.66 (CI 1.15–2.39), *p* = 0.007]. With respect to adult outcomes, after controlling for sex and childhood background factors, those with recurrent disordered gambling were rated by informants as having more financial problems compared to those who had experienced a single episode [*d* = 0.54 (CI 0.19–0.90), *p* = 0.003].

## Discussion

In this population-based birth cohort study, we present novel evidence about the links between disordered gambling and socioeconomic, financial, and legal outcomes in adulthood after taking into account premorbid characteristics (socioeconomic status, IQ, and self-control) of the study members when they were children. We also confirm and extend previous findings that disordered gambling usually has an episodic course, and present new evidence about differences in the precursors and outcomes of recurrent and single-episode disordered gambling.

### Long-term course of disordered gambling

The course of disordered gambling was tracked for longer than in any previous longitudinal study, across four decades from ages 18 to 45. New onsets of disordered gambling occurred (at decreasing frequency) at every wave of the study, even into the fifth decade of life. The most common course of disordered gambling was experiencing just a single episode. These results for subclinical disordered gambling align with evidence based on data from a large cross-sectional survey of gambling disorder (Slutske, 2006).

### Adult life outcomes associated with disordered gambling

Lifetime disordered gambling was significantly associated with socioeconomic disadvantage as indicated by lower occupational and educational attainment. Cohort members with a history of disordered gambling were also more likely to be struggling financially in midlife. Compared to those without a history of disordered gambling, those with disordered gambling had worse credit scores and had spent more time receiving social welfare benefits. These financial difficulties were verified by informants who knew them well, who rated those with disordered gambling as being more likely to be poor money managers and to lack money to make ends meet. Cohort members with a history of disordered gambling also had more criminal convictions. These would all appear to represent consequences of disordered gambling, although with cumulative outcomes such as occupational and educational attainment, it can be difficult to establish temporal precedence in adulthood. We were able to establish temporal precedence because we had information about the study members when they were children, prior to their future gambling involvement.

### Socioeconomic disadvantage and disordered gambling

Children who would go on to develop disordered gambling in adulthood came from more socioeconomically disadvantaged families compared to their peers who would not go on to develop disordered gambling. This is consistent with socioeconomic disadvantage being potentially a causal risk factor for disordered gambling. But after controlling for childhood background factors (including socioeconomic status) the association between disordered gambling and lower occupational and educational attainment at age 45 was still statistically significant (although markedly reduced). These results are consistent with socioeconomic disadvantage also being a potential consequence of disordered gambling. There appears to be a reciprocal relation between socioeconomic status and disordered gambling.

One route by which socioeconomic disadvantage may be related to the development of disordered gambling is through neighborhood characteristics. Those born into more disadvantaged families are more likely to grow up in deprived neighborhoods. Several studies, including one conducted in New Zealand (Pearce, Mason, Hiscock, & Day, 2008), have reported an association between living in a disadvantaged neighborhood and disordered gambling (Martins, Storr, Lee, & Ialongo, 2013; Pearce et al., 2008; Slutske, Deutsch, Statham, & Martin, 2015; Welte et al., 2004). A study of twin pairs discordant for neighborhood disadvantage suggested that this might represent a potentially causal effect (Slutske, Piasecki, Deutsch, Statham, & Martin, 2019). Disadvantaged neighborhoods may generally lead to adverse outcomes via chronic stress and strain or the lack of access to institutional resources (Sampson, Morenoff, & Gannon-Rowley, 2002); they may lead more specifically to disordered gambling via the greater density of gambling venues (Slutske et al., 2015).

### Cognitive abilities and disordered gambling

Children who would go on to develop disordered gambling in adulthood also had lower IQ scores compared to their peers who would not go on to develop disordered gambling. As expected, childhood IQ was also substantially associated with occupational and educational attainment in adulthood (*r* = 0.47 and 0.52, respectively; see online Table S7 in Supplemental Materials). The narrowing of life options may be one mechanism by which lower IQ in childhood might be related to later disordered gambling. In addition, the substantial association between childhood IQ and adult practical financial knowledge (*r* = 0.60, see online Table S7 in Supplemental Materials) suggests that the association between childhood IQ and adult disordered gambling might be mediated in part by a lack of awareness of the basics of money management.

### Self-control, crime, and disordered gambling

Low self-control was associated with disordered gambling in adulthood and was also the strongest predictor, of the three childhood background factors, of adult criminal convictions; the significant association between disordered gambling and adult convictions could be completely explained by low self-control in childhood. This adds to mounting evidence that engaging in criminal activity is not solely a consequence of gambling problems. For example, a retrospective cross-sectional twin study suggested that 61% of the overlap between the adult symptoms of ASPD and disordered gambling could be explained by genetic factors, all of which were onboard in childhood, contributing to the risk for conduct disorder (Slutske et al., 2001). In other words, one reason why antisocial or criminal behavior and gambling problems tend to occur in the same individual is because they have common genetic underpinnings. Here we demonstrate prospectively that a significant risk factor for criminality, low self-control, measured in childhood prior to any engagement in criminal or gambling activities, predicts disordered gambling in adulthood. Individual differences in self-control may represent at least some of the common genetic substrate underlying criminal behavior and disordered gambling.

### Single episode *v.* recurrent disordered gambling

Most individuals who experienced disordered gambling in their lifetime experienced just a single bout, and only a minority experienced multiple episodes, a distinction that has now been incorporated into the DSM. However, not much is known about these two different subgroups or the predictors of disordered gambling recurrence^4^. In the present study, these two groups differed on socioeconomic factors – those with recurrent disordered gambling were more socioeconomically disadvantaged as children and they were more likely to experience financial problems in adulthood compared to those who experienced a single episode of disordered gambling.

### Limitations

This study has at least five limitations. First, the diagnostic assessments of gambling disorder varied across the study waves, even after harmonization. Second, because the past-year prevalences of DSM-5 gambling disorder were low, 2.09% and 0.66% at ages 32 and 45, respectively (DSM-5 diagnostic criteria were not assessed at age 21), we focused on a broad definition of disordered gambling that included individuals with only mild problems. This is consistent with approaches taken in previous research, which has shown that endorsing even a single symptom of disordered gambling is associated with clinically significant outcomes (Slutske, Davis, Lynskey, Heath, & Martin, 2022). In the present study, disordered gambling was associated with important economic and legal outcomes. Stronger associations might have been observed had we been able to focus on more severe cases of gambling disorder. Nonetheless, the measure of disordered gambling was validated by demonstrating a strong association with informant reports of gambling problems at age 45, suggesting that the problems were serious enough to be noticed by significant others. Because gambling pathology is a continuously distributed phenomenon, the insights gained from studying milder forms of gambling disorders will contribute to an understanding of more severe forms.

Third, our findings are specific to a cohort of individuals born in Dunedin, New Zealand, in the early 1970s who were primarily white. Nonetheless, the average per capita gambling loss in New Zealand in 2017 ($454 USD) was quite similar to the United States average ($421 USD; Lett, 2018), and the correlates of disordered gambling appear to generalize across Western nations that differ in their racial/ethnic diversity. For example, the association between neighborhood disadvantage and disordered gambling has been replicated in the US, Great Britain, Canada, Australia, and New Zealand (Slutske et al., 2015).

Fourth, because the measure of lifetime disordered gambling combined assessments from ages 18 to 45, and some of the adult outcomes were cumulative, it was not possible to establish temporal precedence of predictors and outcomes. Even if temporal precedence had been established, however, this would still not have warranted drawing firm conclusions about causality from these observational data.

Fifth, we elected to specifically focus on financial and legal outcomes because they may be especially relevant to gambling disorder and have been less thoroughly studied in the extant literature than have other outcomes, such as mental disorders. Emerging evidence suggests that more sophisticated approaches might be warranted when tackling the issue of co-occurring mental disorders. Future research might revisit the questions addressed in this paper by examining the unfolding of gambling pathology in tandem with other mental disorders (Caspi et al., 2020) or by examining whether disordered gambling predicts important outcomes after accounting for the ‘*p* factor’ (Caspi & Moffitt, 2018).

## Conclusions

This study demonstrates that socioeconomic, financial, and legal outcomes in adulthood are not merely consequences of disordered gambling and that childhood socioeconomic status, IQ, and low self-control prospectively predict disordered gambling in adulthood. This study also presents new evidence relevant to the descriptive epidemiology of disordered gambling. It confirms previous findings that disordered gambling has an episodic course and that most of those affected experience only a single bout. As a result of assessing disordered gambling across six waves of a prospective longitudinal study, this study demonstrates that the proportion of the population that experiences a problem with gambling in their lifetime may have been underestimated based on findings from cross-sectional surveys (Moffitt et al., 2010; Slutske et al., 2003). In addition, lower childhood social class was associated with a recurrent course, reinforcing again that childhood poverty is an important risk factor for disordered gambling.

### Future directions

We have shown that childhood self-control, socioeconomic status, and IQ forecast the development of disordered gambling into midlife. Although socioeconomic status and IQ may be hard to change, there is evidence that self-control may be a more malleable and teachable characteristic (Moffitt, Poulton, & Caspi, 2013; Roberts & Mroczek, 2008). Previously we reported that the relations between low self-control in childhood and socioeconomic, legal, and health outcomes in adulthood were partly mediated by adolescent mistakes, or ‘snares’, including early smoking, school dropout, and teen parenthood (Moffitt et al., 2011). The socioeconomic and legal correlates of disordered gambling identified in our analysis suggest that similar snares may link childhood self-control to adult gambling problems. Therefore, there may be two critical windows for intervening to enhance self-control: (1) early in childhood to prevent adolescent mistakes, and (2) again in adolescence to prevent or ameliorate the consequences of such mistakes (Moffitt et al., 2011).

Although the present study offers the most comprehensive prospective analysis of the epidemiology of disordered gambling to date, additional data are needed to chart the longer-term course and outcomes of disordered gambling into older adulthood. It will be particularly important to continue to follow those individuals who experience recurrent problems. For instance, individuals with recurrent disordered gambling were differentiated from those with single-episode disordered gambling by their experiences of financial problems in midlife. Do such problems also forecast greater financial insecurity at retirement age? Continued longitudinal documentation of the course and consequences of disordered gambling within representative cohorts should be a research priority.

## Footnotes

* The notes appear after the main text.

## Notes

1 Disordered gambling refers to the full continuum of gambling pathology, ranging from experiencing a single DSM-5 symptom of gambling disorder to experiencing all nine symptoms; experiencing four or more symptoms would qualify for a diagnosis of DSM-5 gambling disorder. Because of the focus on past-year disorder, most longitudinal studies, including the present study, have imposed a lower threshold on the continuum of gambling pathology for making a diagnosis, and have termed this ‘disordered gambling’ rather than ‘gambling disorder’.

2 This included both the harmonized and single-item assessments of disordered gambling that were used separately in the stability analyses.

3 The same applied to the small number of study members diagnosed with DSM-5 gambling disorder at ages 32 or 45. Of the 22 study members with gambling disorder who participated at both phases, only 9.1% (*n* = 2) were diagnosed at both phases; 72.7% were only diagnosed at age 32 and 18.2% were only diagnosed at age 45.

4 It is likely that treatment-seeking samples are completely comprised of those with recurrent disordered gambling, whereas community samples would primarily include those who have experienced only a single episode.

## Supplementary material

For supplementary material accompanying this paper visit https://doi.org/10.1017/S0033291722003051. click here to view supplementary material
