# Supplementary material for: Disordered gambling in a longitudinal birth cohort: from childhood precursors to adult life outcomes
Source: Psychol Med. 2022 Oct 18;53(12):5800–8. doi: 10.1017/S0033291722003051 (PMC10482703; doi:10.1017/S0033291722003051)
Supplement: Supplementary file 1 [file S0033291722003051sup001.docx]

**SUPPLEMENTAL MATERIALS**

***for***

**Disordered Gambling in a Longitudinal Birth Cohort:**

**From Childhood Precursors to Adult Life Outcomes**

Wendy S. Slutske, Leah S. Richmond-Rakerd, Thomas M. Piasecki,

Sandhya Ramrakha, Richie Poulton, Terrie E. Moffitt, & Avshalom Caspi

**Contents**

Representativeness of Dunedin Cohort

Table S1. Harmonization of gambling disorder assessments at Phases 21, 32, and 45

Description of measures of childhood background factors

Table S2. Description of the childhood self-control composite

Table S3. Measures of adult outcomes

Table S4. Course of single item disordered gambling across five study phases.

Table S5. Course of any gambling involvement across three study phases.

Table S6. Associations between disordered gambling at six study phases.

Table S7. Correlations among childhood background factors and participant sex with adult outcomes.

**Representativeness of the Dunedin cohort**

On adult health, the cohort matches the New Zealand National Health and Nutrition Survey on key health indicators (e.g., body mass index, smoking, visits to the doctor) and matches the New Zealand Census of people the same age on educational attainment (Richmond-Rakerd et al., 2020).

**References**

Richmond-Rakerd, L.S., D’Souza, S., Andersen, S.H., Hogan, S., Houts, R.M., Poulton, R., . . . Moffitt, T.E. (2020). Clustering of health, crime and social-welfare inequality in 4 million citizens from two nations. Nature Human Behaviour, 4:255-264.

| **Table S1. Harmonization of gambling disorder assessments at Phases 21, 32, and 45** | | | |
| --- | --- | --- | --- |
| Symptom | Phase 21 | Phase 32 | Phase 45 |
| 1) financial problems | How many times in the last year have you gambled with money that you or your family needed for living expenses (cut-off > 0) | In the past year, has gambling or betting ever been a problem for you? For example, have you bet or gambled money your family needed? Borrowed money, sold property or gone into overdraft to pay for gambling? (SRD) | Has your gambling caused any financial problems for you or your household? (PSGI) |
| 2) chasing losses | If you lose at gambling, do you gamble again to try to win back the money? (SOGS) | Has there been a period when, if you lost money gambling one day, you would return another day to get even? (DSM-IV) | When you gambled, did you go back another day to try to win back the money you lost? (PGSI) |
| 3) spending more time or money than intended | Have you spent more time or money at gambling than you intended to? (SOGS) | I have spent more money during a gambling session than was my original intention (cut-off > 2) (SLUGS)  Or  I have spent more time in a gambling session than was my original intention (cut-off > 2) (SLUGS)  Or  Have there been periods when you needed to gamble with increasing amounts of money or with larger bets than before in order to get the same feeling of excitement. (DSM-IV) | Have you needed to gamble with larger amounts of money to get the same feeling of excitement? (PGSI) |
| 4) borrowing money | Have you ever borrowed money, sold property, or gone into overdraft to pay for your gambling? (SOGS) | Have you needed to ask family members or anyone else to loan you money or otherwise bail you out of a desperate money situation that was largely caused by your gambling? (DSM-IV) | Have you borrowed money or sold something to get money to gamble? (PGSI) |
| 5) hiding evidence or lying about gambling | Have you hidden lottery tickets, betting slips, money or other signs of gambling so that your family or partner would not know? (SOGS) | Have you lied to family members or others about how much you gamble or how much money you lost on gambling? (DSM-IV) | Do you lie to family members or to others to hide how much you are gambling? (DSM-IV) |
| Notes: SRD = self-reported delinquency scale, PGSI = Problem Gambling Severity Index, SOGS = South Oaks Gambling Screen, DSM-IV = Diagnostic and Statistical Manual, Fourth Revision, SLUGS = Sydney Laval Universities Gambling Screen. | | | |

**Description of measures of childhood background factors**

Social class. The socioeconomic statuses of cohort members’ families were measured using a six-point scale that assessed parents’ occupational statuses, defined based on average income and educational levels derived from the New Zealand Census (Elley & Irving, 1976). Parents’ occupational statuses were assessed when participants were born and again at subsequent assessments up to age 15 years. The highest occupational status of either parent was averaged across the childhood assessments (Poulton et al., 2002).

General intelligence (IQ). The Wechsler Intelligence Scale for Children-Revised (WISC-R (Wechsler, 1974)) was administered to the study members at ages 7, 9, and 11 years. IQ scores for the three ages were averaged and standardized (M=100, SD=15).

Self-control. Children’s self-control during their first decade of life was measured using a multi-occasion/multi-informant strategy. Briefly, the nine measures of childhood self-control in the composite include observational ratings of children’s lack of control; parent and teacher reports of impulsive aggression; and parent, teacher, and self-reports of hyperactivity, lack of persistence, inattention, and impulsivity (See Table S2).

**References**

Elley, W.B., & Irving, J.C. (1976). Revised socio-economic index for New Zealand. New Zealand Journal of Educational Studies, 7:153-167.

Poulton, R., Caspi, A., Milne, B.J., Thomson, W.M., Taylor, A., Sears, M.R., & Moffitt, T.E. (2002). Association between children’s experience of socioeconomic disadvantage and adult health: a life-course study. Lancet, 360:1640-1645.

Wechsler, D. (1974). Manual for the Wechsler Intelligence Scale for Children-Revised. New York, NY: Psychological Corporation.

| **Table S2. Description of the childhood self-control composite** | | | |
| --- | --- | --- | --- |
| - At ages 3 and 5, each study child participated in a testing session involving cognitive and motor tasks. The children were tested by examiners who had no knowledge of their behavioral history. Following the testing, each examiner rated the child’s lack of control in the testing session (Caspi et al., 1995). - At ages 5, 7, 9, and 11, parents and teachers completed the Rutter Child Scale (RCS (Elander & Rutter, 1996)), which included items indexing impulsive aggression and hyperactivity. - At ages 9 and 11, the RCS was supplemented with additional questions about the children’s lack of persistence, inattention, and impulsivity (McGee et al., 1992). - At age 11, children were interviewed by a psychiatrist and reported about their symptoms of hyperactivity, inattention, and impulsivity (Anderson et al., 1987)   The nine measures of self-control in childhood were all similarly positively and significantly correlated. Based on principal components analysis, the standardized components were averaged into a single composite score (M=0, SD=1) with excellent internal reliability (α=.86 (Moffitt et al., 2011)); the first component in a principal components analysis accounted for 51% of the variance. | | | |
| **Summary of measures included** | | | |
| **Measure** | **Age(s) assessed** | **Source** | **Item content** |
| Lack of control | 3, 5 | Observer | Labile, low frustration tolerance, lack of reserve, resistance, restless, impulsive, requires attention, brief attention to task, lacks persistence in reaching goals |
| Impulsive aggression | 5, 7, 9, 11 | Parent, teacher | Flies off handle, fights |
| Hyperactivity | 5, 7, 9, 11 | Parent, teacher | Runs and jumps about, cannot settle, has short attention span |
| Hyperactivity | 9, 11 (additional items) | Parent, teacher | “On the go” as if “driven by a motor”, difficulty sitting still |
| Lack of persistence | 9, 11 | Parent, teacher | Fails to finish tasks, easily distracted, difficulty sticking to activity |
| Impulsivity | 9, 11 | Parent, teacher | Acts before thinking, has difficulty awaiting turn, shifts excessively between activities |
| Hyperactivity | 11 | Self | Fidgety, restless |
| Inattention | 11 | Self | Difficulty paying attention, trouble sticking to a task |
| Impulsivity | 11 | Self | Difficulty waiting turn, talking while others are still talking |

**References**

Anderson, J.C., Williams, S., McGee, R., & Silva, P.A. (1987) DSM-III disorders in preadolescent children. Prevalence in a large sample from the general population. Archives of General Psychiatry, 44, 69-76.

Caspi, A., Henry, B., McGee, R.O., Moffitt, T.E., & Silva, P.A. (1995). Temperamental origins of child and adolescent behavior problems: from age 3 to age 15. Child Development, 66:55-68.

Elander, J., & Rutter, M. (1996). Use and development of the Rutter parents' and teachers' scales. International Journal of Methods in Psychiatric Research, 6:63-78.

McGee, R., Feehan, M., Williams, S., & Anderson, J. (1992). DSM-III disorders from age 11 to age 15 years. Journal of the American Academy of Child & Adolescent Psychiatry, 31(1), 50-59.

| **Table S3. Measures of adult outcomes** | |
| --- | --- |
| **Outcome** | **Description** |
| **Socioeconomic** |  |
| Occupational attainment | Study members’ occupational attainment at age 45 was measured according to the New Zealand Socio-Economic Index 2006 (NZSEI-06; Milne, Byun, & Lee, 2013), a six-group occupation-based measure of socioeconomic status. Homemakers and others not working in the past year were assigned the socioeconomic status of their most recent occupation, as reported at age 38. Study members who had been out of the labor force since age 32 were assigned the socioeconomic status of their partner; if they did not share a household with a partner, their socioeconomic status was assigned based on their education level. |
| Educational attainment | Study members’ educational attainment was measured as the highest degree completed by the age-45 assessment. |
| **Financial** |  |
| Credit score | Credit scores were acquired at the age-45 assessment phase from the Equifax/Veda Company. The credit score algorithm is proprietary. Scores are based on five-year histories of consumer credit activity and include information on factors including the number and types of credit applications and inquiries, residential stability, and adverse information such as payment defaults and judgments. Factors such as race, national origin, marital status, occupation, salary, employment history, and medical or academic records are not included in Equifax/Veda scoring. Of the 938 study members who participated in the age-45 assessment, 724 consented to a credit-rating search and were credit-active in New Zealand in the last five years. The majority of study members who were not active resided overseas; we imputed age-45 credit scores for these individuals based on their scores at the prior assessment wave (age 38 years). Six study members were flagged by Equifax/Veda as insolvent at phase 45; we assigned them a score of 66 (one point less than the lowest score among study members with a credit score). There were 891 total study members with credit-score data. |
| Informant reported financial problems | At age 45, study members were asked to nominate someone “who knew them well” (the majority of reports were provided by friends, partners, and family members). Full details of the Dunedin Study informant rating system are provided elsewhere (Moffitt et al, 2002). Informants rated the study member on two items: “poor money manager” using a three-point scale (0=not a problem, 1=bit of a problem, 2=yes, a problem), and “lacks enough money to make ends meet” (0=not a problem, 1=bit of a problem, 2=yes, a problem). Responses for each item were averaged across informants, and then the two items were averaged. |
| Practical financial knowledge | Study members’ practical financial knowledge at age 45 was indexed by two scales:  Multiple-choice assessment. Participants were administered a six-item multiple-choice assessment of their understanding of different financial principles, including items adapted from the Organization for Economic Co-operation and Development/International Network on Financial Education Toolkit (OECD, 2013). Principles included those related to mortgages/loans, inflation, interest, and risk and return. The number of correct responses was summed to form a scale (range=0-6, α=0.64).  Open-ended interview. Participants were interviewed about their understanding of different financial principles, with an open-ended response format: “What is the advantage of paying off your credit card balance each month?”; “Why do some people spread out investments and savings in different types of schemes?”; “How does the inflation rate affect the money you keep in a savings account?”; “Why do some people take fewer financial risks as they get older?”; and “What is the advantage to starting retirement savings when a person is young?” Using standardized scoring procedures, four trained raters (two raters per interview) coded responses on a scale from 0 to 2, with 0 indicating no understanding of the financial principle, 1 indicating moderate understanding, and 2 indicating good understanding (interrater reliability=0.97). For instance, in response to the question “What is the advantage of paying off your credit card balance each month?”, the following responses were coded as 0, 1, and 2, respectively: “To avoid penalties,” “A better credit rating,” and “To avoid interest.” Scale scores were computed by summing across the items and then averaging across raters (range=0-10).  The multiple-choice and open-ended scales were correlated (r=0.63, p<.0001). The Practical Financial Knowledge measure was computed by standardizing (M=0, SD=1) and averaging the multiple-choice and open-ended scales. |
| Unemployment | At ages 26, 32, 38, and 45 years, study members reported on their spells of unemployment using Life History Calendars (Caspi et al., 1996). We combined information across assessments to create a measure of study members’ total number of months of unemployment since age 26. |
| Social welfare benefits | At age 45 we collected information about social-welfare benefits received – including the total number of months study members had spent on benefits – from administrative records maintained by the New Zealand Ministry of Social Development. Data were available from January 1993 onward. |
| **Legal** |  |
| Adult convictions | We collected information about study members’ criminal-conviction histories using administrative records maintained by the New Zealand Ministry of Justice. We measured adult convictions accumulated up to age 45 years. |

**References**

Caspi, A., Moffitt, T.E., Thornton, A., . . . Silva, P.A. (1996). The Life History Calendar: a research and clinical assessment method for collecting retrospective event-history data. International Journal of Methods in Psychiatric Research, 6101-6114.

Milne, B. J., Atkinson, J., Blakely, T., Day, H., Douwes, J., Gibb, S., ... & Teng, A. (2019). Data resource profile: The New Zealand Integrated Data Infrastructure (IDI). International Journal of Epidemiology, 677–677.

Milne, B.J., Byun, U., & Lee, A. (2013). New Zealand socio-economic index 2006. Wellington, NZ: Statistics New Zealand.

Moffitt, T. E., Caspi, A., Harrington, H., & Milne, B. J. (2002). Males on the life-course-persistent and adolescence-limited antisocial pathways: Follow-up at age 26 years. Development and Psychopathology, 14(1), 179-207.

OECD (2013). OECD/INFE toolkit to measure financial literacy and financial inclusion: guidance, core questionnaire and supplementary questions. Retrieved from <https://www.oecd.org/daf/fin/financial-education/TrustFund2013_OECD_INFE_toolkit_to_measure_fin_lit_and_fin_incl.pdf>

| **Table S4. Course of single item disordered gambling across five study phases.** | | | | | | | | | | | |
| --- | --- | --- | --- | --- | --- | --- | --- | --- | --- | --- | --- |
|  | Full sample | 1 or more episodes | 2 or more episodes |  | Study Phase (age) | | | | |  |  |
|  | % | % | % | *n* | 18 | 26 | 32 | 38 | 45 | % | *n* |
| No disorder | 94.80 | --- | --- | 856 | - | - | - | - | - | 96.61 | 856 |
| Single episode | 3.65 | 70.21 | --- | 33 | + | - | - | - | - | 1.22 | 11 |
|  |  |  |  |  | - | + | - | - | - | 1.00 | 9 |
|  |  |  |  |  | - | - | + | - | - | 0.44 | 4 |
|  |  |  |  |  | - | - | - | + | - | 0.66 | 6 |
|  |  |  |  |  | - | - | - | - | + | 0.33 | 3 |
| Recurrent/  persistent | 1.55 | 29.79 | 2 episodes:  64.29 | 14 | - | + | + | - | - | 0.22 | 2 |
|  |  |  |  |  | - | + | - | + | - | 0.11 | 1 |
|  |  |  |  |  | - | + | - | - | + | 0.11 | 1 |
|  |  |  |  |  | - | - | + | + | - | 0.22 | 2 |
|  |  |  |  |  | - | - | + | - | + | 0.22 | 2 |
|  |  |  |  |  | - | - | - | + | + | 0.11 | 1 |
|  |  |  | 3 episodes:  21.43 |  | - | + | + | + | - | 0.11 | 1 |
|  |  |  |  |  | - | + | + | - | + | 0.11 | 1 |
|  |  |  |  |  | - | + | - | + | + | 0.00 | 0 |
|  |  |  |  |  | - | - | + | + | + | 0.11 | 1 |
|  |  |  | 4 episodes:  14.29 |  | - | + | + | + | + | 0.22 | 2 |
| Note: item was identical at all phases but 18, individuals who endorsed the age 18 item did not endorse the item at the subsequent four ages, minus = absence of disordered gambling, plus = presence of disordered gambling. | | | | | | | | | | | |

| **Table S5. Course of any gambling involvement across three study phases.** | | | | | | | |
| --- | --- | --- | --- | --- | --- | --- | --- |
|  | % | n | 21 | 32 | 45 | % | n |
| No gambling | 4.42 | 38 | - | - | - | 4.42 | 38 |
| Single time point | 10.13 | 87 | + | - | - | 6.40 | 55 |
|  |  |  | - | + | - | 2.44 | 21 |
|  |  |  | - | - | + | 1.28 | 11 |
| Multiple time points | 85.45 | 734 | + | + | - | 9.55 | 82 |
|  |  |  | + | - | + | 7.92 | 68 |
|  |  |  | - | + | + | 4.54 | 39 |
|  |  |  | + | + | + | 63.45 | 545 |
| Note: minus = absence of any gambling, plus = presence of any gambling | | | | | | | |

| **Table S6. Associations between disordered gambling at six study phases (the three bolded waves at ages 21, 32, and 45 included a more comprehensive 5-item harmonized disordered gambling assessment, the nonbolded waves include a single-item assessment).** | | | | | | |
| --- | --- | --- | --- | --- | --- | --- |
|  | Study Phase (age) | | | | | |
| Study Phase (age) |  | 18 | **21** | 26 | **32** | 38 |
|  | **21** | .80 |  |  |  |  |
|  | 26 | *nc* | .71 |  |  |  |
|  | **32** | *nc* | .60 | .97 |  |  |
|  | 38 | *nc* | .71 | .92 | .96 |  |
|  | **45** | *nc* | .70 | .95 | .91 | .96 |
| Note: Cell entries are gamma coefficients. nc = not calculable due to small cell sizes. All gamma coefficients are significant at *p* < .006 | | | | | | |

| **Table S7. Correlations among childhood background factors and participant sex and with adult outcomes.** | | | | |
| --- | --- | --- | --- | --- |
| **Childhood background factors** | | | | |
|  | 1 | 2 | 3 | 4 |
| 1. Childhood socioeconomic status | --- |  |  |  |
| 2. Childhood IQ | **.41** | --- |  |  |
| 3. Childhood low self-control | **-.26** | **-.45** | --- |  |
| 4. Participant sex (0=female, 1=male) | .00 | .04 | **.25** | --- |
| **Adult outcomes** | | | | |
| Disordered gambling (main outcome) | **-.11** | **-.08** | **.08** | **.20** |
| Gambling versatility, 45 | **-.12** | **-.08** | .00 | .05 |
| Informant reported gambling problems, 45 | **-.08** | -.05 | **.08** | .04 |
| Occupational attainment, 45 | **.35** | **.47** | **-.32** | **-.10** |
| Educational attainment, 45 | **.40** | **.52** | **-.42** | **-.12** |
| Credit score, 45 | **.17** | **.13** | **-.15** | **-.10** |
| Practical financial knowledge, 45 | **.35** | **.60** | **-.39** | **.14** |
| Months unemployed, 26-45 | -.06 | **-.16** | **.17** | .02 |
| Months social welfare benefits, thru 45 | **-.19** | **-.34** | **.31** | -.05 |
| Informant reported financial problems, 45 | -.05 | **-.14** | **.17** | .05 |
| Adult convictions, thru 45 | **-.14** | **-.15** | **.23** | **.15** |
| Note: All correlations are sex adjusted; bolded estimates are statistically significant at p<.05. | | | | |
